# Comparative study of the effects of cigarette smoke versus next-generation tobacco and nicotine product extracts on inflammatory biomarkers of human monocytes

**DOI:** 10.1007/s00424-023-02809-9

**Published:** 2023-04-21

**Authors:** Sindy Giebe, Melanie Brux, Anja Hofmann, Frazer Lowe, Damien Breheny, Henning Morawietz, Coy Brunssen

**Affiliations:** 1 Division of Vascular Endothelium and Microcirculation, Department of Medicine III, University Hospital Carl Gustav Carus Dresden, Technische Universität Dresden, Fetscherstr. 74, D-01307 Dresden, Germany; 2 B.A.T. (Investments) Limited, Regents Park Road, Millbrook, Southampton, SO15 8TL UK

**Keywords:** 3R4F aqueous cigarette smoke extract (3R4F AqE), Next-generation tobacco and nicotine products (NGP), Heated tobacco product (HTP), Electronic cigarette (e-cig), Monocyte adhesion molecules, Inflammatory biomarkers, Endothelial dysfunction

## Abstract

Monocytes exhibiting a pro-inflammatory phenotype play a key role in adhesion and development of atherosclerotic plaques. As an alternative to smoking, next-generation tobacco and nicotine products (NGP) are now widely used. However, little is known about their pro-inflammatory effects on monocytes. We investigated cell viability, anti-oxidant and pro-inflammatory gene and protein expression in THP-1 monocytes after exposure to aqueous smoke extracts (AqE) of a heated tobacco product (HTP), an electronic cigarette (e-cig), a conventional cigarette (3R4F) and pure nicotine (nic). Treatment with 3R4F reduced cell viability in a dose-dependent manner, whereas exposure to alternative smoking products showed no difference to control. At the highest non-lethal dose of 3R4F (20%), the following notable mRNA expression changes were observed for 3R4F, HTP, and e-cig respectively, relative to control; *HMOX1* (6-fold, < 2-fold, < 2-fold), *NQO1* (3.5-fold, < 2-fold, < 2-fold), *CCL2* (4-fold, 3.5-fold, 2.5-fold), *IL1B* (4-fold, 3-fold, < 2-fold), *IL8* (5-fold, 2-fold, 2-fold), *TNF* (2-fold, 2-fold, < 2-fold) and *ICAM*1 was below the 2-fold threshold for all products. With respect to protein expression, IL1B (3-fold, < 2-fold, < 2-fold) and IL8 (3.5-fold, 2-fold, 2-fold) were elevated over the 2-fold threshold, whereas CCL2, TNF, and ICAM1 were below 2-fold expression for all products. At higher doses, greater inductions were observed with all extracts; however, NGP responses were typically lower than 3R4F. In conclusion, anti-oxidative and pro-inflammatory processes were activated by all products. NGPs overall showed lower responses relative to controls than THP-1 cells exposed to 3R4F AqE.

## Introduction

Tobacco smoking is a key stimulus in the development of atherosclerosis [29, 42, 50]. A crucial step in the initiation of atherosclerosis is the development of an endothelial dysfunction. During this early stage, the side of the atherosclerotic lesion shows a pro-inflammatory phenotype characterized by changes in expression profile, increased inflammatory biomarkers, impaired endothelial barrier function, and adhesion of monocytes to endothelial cells. Activation and adhesion of monocytes to the endothelium therefore play a major role in atherogenesis [11]. Several studies indicated an atherosclerosis-related gene expression profile of endothelial cells as well as of monocytes in the presence of the main behavioural risk factor smoking [3, 34, 41, 73, 77]. Independent stimulation with aqueous smoke extract (AqE) of conventional reference cigarettes (3R4F) revealed cytotoxic, oxidative, and pro-inflammatory effects on monocytes, shown in an own previous study [9].

Smoking leads to oxidative stress and mediates the development of reactive oxygen species (ROS), which are key mediators for smoke-induced vascular inflammation during atherogenesis. The family of NADPH oxidases is one of the major sources of cellular ROS. In monocytes, the NADPH oxidase isoform 2 (NOX2), also known as cytochrome b-245 beta chain (CYBB), is the main source and marker of oxidative stress which produces superoxide anions (O_2_^−^) [8, 32, 45]. The production of superoxide anions (O_2_^−^) by NOX2 has been shown to be involved in the development of endothelial dysfunction and atherosclerosis [43]. As protection against these oxidative damages, cells have very effective anti-oxidative repair and defence mechanisms [5, 17, 20, 65, 72]. Transcription factor NRF2 (NFE2L2) is the main mediator of cellular adaptation to oxidative stress. NRF2 is activated by oxidative stimuli and acts to prevent intracellular redox imbalance [22]. After activation, NRF2 binds to antioxidant response elements (ARE) in regulatory regions of detoxifying and antioxidant target genes, such as heme oxygenase (decycling) 1 (*HMOX1*) and NAD(P)H dehydrogenase (quinone 1) (*NQO1*) and modulates their transcription [13, 44]. The process of vascular inflammation is accompanied by a pro-inflammatory phenotype which is characterized by an activation of key markers like intercellular adhesion molecule 1 (ICAM-1), vascular cell adhesion molecule 1 (VCAM-1), selectin E (SELE), and platelet and endothelial cell adhesion molecule 1 (CD31) [16, 25, 33, 39]. Acute exposure to cigarette smoke induces an inflammatory response with early activation of inflammatory cells and a corresponding release of various inflammatory mediators [42]. C-C motif chemokine ligand 2 (CCL2/MCP1) is a monocyte-specific surface marker that accelerates atherosclerosis and shows an increased expression in atherosclerotic lesions [2, 40]. In addition, various inflammatory cytokines such as interleukin 1-beta (IL1B), interleukin 8 (CXCL8), and tumour necrosis factor-alpha (TNF) show in vivo as well as in vitro an elevated expression and secretion under smoking conditions [14, 49, 74].

Over the last years, we have intensively studied the effects of smoking on the progress of atherosclerosis on a cellular level. First, we started investigating the impact of conventional smoking not only on endothelial cells but also on human monocytes. These cell types are constantly exposed and directly affected by circulating smoke-mediated toxicants and reactive oxygen species in the bloodstream and mediated important steps in the initiation and first steps of atherogenesis [9, 26].

One strategy to reduce the harmful side effects of tobacco smoking is the development of next-generation tobacco and nicotine products (NGP) [63]. Over the last years, electronic cigarettes (e-cig) for inhalation of nicotine as well as heated tobacco products (HTP), based on heating rather than burning tobacco, were established [30, 61]. These newly developed tobacco and nicotine products are considered a “safer alternative” to conventional cigarettes with less harmful effects [37, 47, 48, 51, 62, 69, 71]. However, little is known about the actual harmful potential of next-generation tobacco and nicotine products (NGP) in terms of cell viability, oxidative stress, and the switch of cells to a pro-inflammatory phenotype in the vasculature. Therefore, we investigated the effects of NGPs on endothelial function [27] as well as on monocyte function which are presented in the following study.

## Materials and methods

The present study is a follow-up study. Therefore, only brief information is provided in the following sections. For detailed descriptions of the material and methods used in this study, please refer to our previous publications [9, 26, 27].

### Cell culture

Human monocytic THP-1 cells (ATCC# TIB-202) were used in this study. Cells were cultured for a maximum period of 8 weeks in RPMI-1640 (supplemented with 10% foetal calf serum, 100,000 U/L penicillin, 100 mg/L streptomycin). Every 2–3 days, THP-1 cells were split (ratio 1:8) in fresh culture media for further cultivation [9, 12, 70].

### Aqueous smoke extract production and stimulation

3R4F reference cigarettes, a heated tobacco product (HTP; iQOS) and a nicotine product (e-cig; VYPE), were used in this study (for detailed information please see Table 1). All tested products were machine-puffed following the corresponding puffing regime (Table 1). All aqueous smoke extracts (AqE) were generated by bubbling evolving smoke through a phenol red-free M199 medium. This procedure provided a stock solution of 100 %. Nicotine concentrations are shown in Table 2 [7, 27].

**Table 1:** Specifications of test products and parameters for aqueous smoke extracts (AqE) generation

	**3R4F**	**e-cig**	**HTP**	**nic**
**Product**	3R4F reference cigarette	VYPE ePen 2.0	iQOS	Nicotine solution
**Manufacturer**	University of Kentucky, Lexington, KY, USA	British American Tobacco, London, UK	Philip Morris International, Lausanne, Switzerland	Sigma-Aldrich, Munich, Germany
**Consumables**	n/a	Blended tobacco e-liquid	Marlboro Essence HeatSticks	n/a
**Nicotine content**	0.73 mg/cig	18.00 mg/ml	0.37 mg/stick	1.00 g/ml
**Puff regime**	HCI	CRM No. 81	HCIm	n/a
**Puff volume**	55 ml	55 ml	55 ml	n/a
**Puff duration**	2 s	3 s	2 s	n/a
**Puff frequency**	30 s	30 s	30 s	n/a
**Puff profile**	Bell	Square	Bell	n/a
**Vent blocking**	Yes	n/a	No	n/a
**Number of puffs**	10 puffs	10 puffs	10 puffs	n/a
**Capture solvent**	M199 w/o phenol red	M199 w/o phenol red	M199 w/o phenol red	n/a
**Volume of capture solvent**	20 ml	20 ml	20 ml	n/a
**Settings**	90° vaping angle	High power (4.4 W); 4-s button activated (1-s pre-activation followed by 3-s activation during puffing); 45° vaping angle	4-s button activated followed by 20-s pre-heating (no button pressed) prior 1st puff; 90° vaping angle	n/a

**Table 2:** Amount of nicotine in different concentrations of aqueous extracts of tobacco and nicotine products and pure nicotine solution (control)

**Concentration [%]**	**Nicotine amount [μg/ml]**			
	**3R4F**	**e-cig**	**HTP**	**nic**
10.0	0.56	0.49	0.30	0.56
20.0	1.12	0.98	0.61	1.12
30.0	1.68	1.47	0.91	1.68
40.0	2.25	1.96	1.21	2.25
50.0	2.81	2.45	1.52	2.81
60.0	3.37	2.93	1.82	3.37
70.0	3.93	3.42	2.13	3.93
80.0	4.49	3.91	2.43	4.49
88.3	4.96	4.32	2.68	4.96
100.0	5.61	4.89	3.04	5.61

Statistics: *n* ≥ 9

THP-1 cells were exposed to increasing AqE dosages (0–88.3 %) of 3R4F, HTP, e-cig, and pure nicotine (control) for the indicated time period. Each sample was accompanied by a control without additional stimulation (time-matched controls) [27].

### Cell viability assay

After cultivation, monocytes were seeded in a 96-well plate (25,000 cells per well), stimulated with the different test substances (0-88.3 % for 24h). Cell viability assay (CellTiter-Glo Luminescent Cell Viability Assay, Promega) was performed and luminescence emission was detected [9, 26, 27].

### Real-time PCR

Monocytes were seeded (70,000 per well / 24-well plate), stimulated with AqE of 3R4F, HTP, e-cig, or pure nicotine (0-88.3 % for 24h). After treatment, total RNA was isolated (High Pure RNA Isolation Kit/Roche Diagnostics) and reverse transcription of mRNA into cDNA was performed (SuperScript II Reverse Transcriptase/Thermo Fisher Scientific). For qPCR, GoTaq qPCR Master Mix (Promega) was used with gene-specific primers (Table 3). Each run was accompanied by melt-curve analysis to ensure a single amplified product. Data were evaluated by using a mathematical model of relative expression ratio in real-time PCR under constant reference gene expression [9, 26, 27, 53].

**Table 3:** Primers used for analysis of human gene expression by real-time PCR

**Target gene**	**Primer**	**sequence (5′ → 3′)**
***POLR2A***	Sense	ACCTGCGGTCCACGTTGTGT
	Antisense	CCACCATTTCCCCGGGATGCG
***NRF2***	Sense	CCCAATTCAGCCAGCCCAGC
	Antisense	AACGGGAATGTCTGCGCCAA
***HMOX1***	Sense	CGGATGGAGCGTCCGCAACC
	Antisense	TCACCAGCTTGAAGCCGTCTCG
***NQO1***	Sense	CCCCGGACTGCACCAGAGC
	Antisense	CTGCAGCAGCCTCCTTCATGGC
***NOX2***	Sense	GCTGTTCAATGCTTGTGGCT
	Antisense	TCTCCTCATCATGGTGCACA
***ICAM1***	Sense	ACCATGGAGCCAATTTCTCG
	Antisense	GCGCCGGAAAGCTGTAGATG
***CCL2***	Sense	CTCTCGCCTCCAGCATGAAA
	Antisense	AGGTGACTGGGGCATTGATT
***IL1B***	Sense	CTCTTCAGCCAATCTTCATTGCTC
	Antisense	TAGGGCCATCAGCTTCAAAGAA
***IL8***	Sense	GGAGAAGTTTTTGAAGAGGGCTGAG
	Antisense	GAATCTTGTATTGCATCTGGCAACC
***TNF***	Sense	CCTGCTGCACTTTGGAGTGA
	Antisense	CTTGTCACTCGGGGTTCGAG

Abbreviations: *POLR2A*, RNA polymerase II subunit A; *NRF2*, nuclear factor erythroid 2-related factor 2; *HMOX1*, heme oxygenase (decycling) 1; *NQO1*, NAD(P)H dehydrogenase (quinone 1); *NOX2*, NADPH oxidase 2; *ICAM-1*, intercellular adhesion molecule-1; *CCL2*, chemokine (C-C motif) ligand 2/*MCP1*, monocyte-chemoattractant protein-1; *IL1B*, interleukin 1 beta; *IL8*, interleukin 8 / *CXCL8*, chemokine (C-X-C Motif) ligand 8; *TNF*, tumour necrosis factor

### Multiplex enzyme-linked immunosorbent assay

Monocytes were seeded (70,000 per well/24-well plate), stimulated with AqE of 3R4F, HTP, e-cig, or pure nicotine (0-88.3 % for 24h). Whole-cell extracts were prepared (RIPA Buffer/Cell Signalling) and protein concentration was determined (BCA Protein Assay Reagent/Perbio Science) [26, 27].

Inflammatory biomarkers were quantified using the electrochemiluminescence-based Mesoscale Discovery (MSD) V-Plex assay platform (MesoScale Discovery, Gaithersburg, MD, USA). Protein samples were analysed using the Vascular Injury Panel 2 (human) Kit (ICAM1), Chemokine Panel 1 (human) Kit (CCL2), and Proinflammatory Panel 1 (human) Kit (IL1B, IL8, TNF).

Assay was performed briefly as follows: preparation of analyte detection plates following the manufacturer’s instructions; adding serial dilution of calibrators and the test samples in duplicates to the wells; incubation under shaking for 1 h; preparing SULFO-TAG labelled detection antibody solutions; washing plates; adding respective detection antibody mixture to each well; incubation under shaking for 1 h; washing plates; adding Read buffer to each well. Finally, plates were detected using the MSD Plate reader (MESO QuickPlex SQ 120).

## Results

### 3R4F, but not NGPs mediate cytotoxic effects on cell viability of human monocytes

**Fig. 1:**
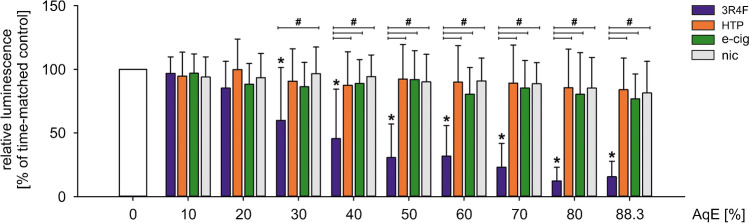
3R4F, but not NGPs mediate cytotoxic effects on cell viability of human monocytes. Evaluation of monocyte cell viability under static conditions and indicated dosages of aqueous extracts using CellTiter-Glo Luminescent Cell Viability Assay. THP-1 monocytes were stimulated with aqueous extracts of indicated tobacco products in a concentration range from 10 to 88.3% for 24 h. Data are shown as mean (% of time-matched controls) ± SD. Statistics: one-way ANOVA, *p < 0.05 vs. time-matched control, #*p* < 0.05, *n* ≥ 9

First, the putative harmful effects of different types of aqueous extracts (AqE) produced were studied. Human THP-1 monocytes were exposed to increasing dosages of 3R4F, HTP, e-cig, and nic ranging from 10 to 88.3 % for 24 h. Treatment with 30% or higher dosages of 3R4F reduced monocytic cell viability in a dose-dependent manner compared to unstimulated controls. All other test reagents did not show differences in cell viability compared to controls (Fig. 1).

### Antioxidative signalling pathways are activated by AqEs of tobacco and nicotine products

**Fig. 2:**
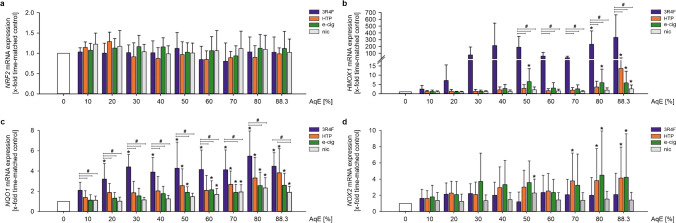
Antioxidative signalling pathways are activated by AqEs of tobacco and nicotine products. mRNA expression of NRF2 (**a**), HMOX1 (**b**), NQO1 (**c**), and NOX2 (**d**) under static conditions was determined after dose-dependent stimulation for 24 h. Data are shown as mean (*x*-fold time-matched controls) ± SD. Statistics: one-way ANOVA *p < 0.05 vs. time-matched control, #*p* < 0.05, *n* ≥ 4

The Main mediator of cellular adaptation to oxidative stress is the NRF2 system. Activation of this system is a critical response of cells to survive oxidative stress situations. Exposure of THP-1 cells to test substances in an increasing dosage activated the NRF2 antioxidant defence system. While mRNA expression of the transcription factor *NRF2* itself remained unchanged under AqE stimulation (Fig. 2a), expression of NRF2 target genes *HMOX1* and *NQO1* revealed a dose-dependent induction (Fig. 2b, c). Here, the responses to alternative tobacco and nicotine products were typically lower than 3R4F stimulation but still showed dose-dependent activation of the NRF2 antioxidant defence system.

NADPH oxidase 2 (*NOX2*) as a main marker of oxidative stress in monocytes, showed a slight upregulation on mRNA level after treatment with 3R4F, HTP, and e-cig. Exposure to pure nicotine solution did not affect *NOX2* in a constant manner (Fig. 2c).

### Adhesion molecule expression is induced after exposure to smoke extracts of all tested products

**Fig. 3:**
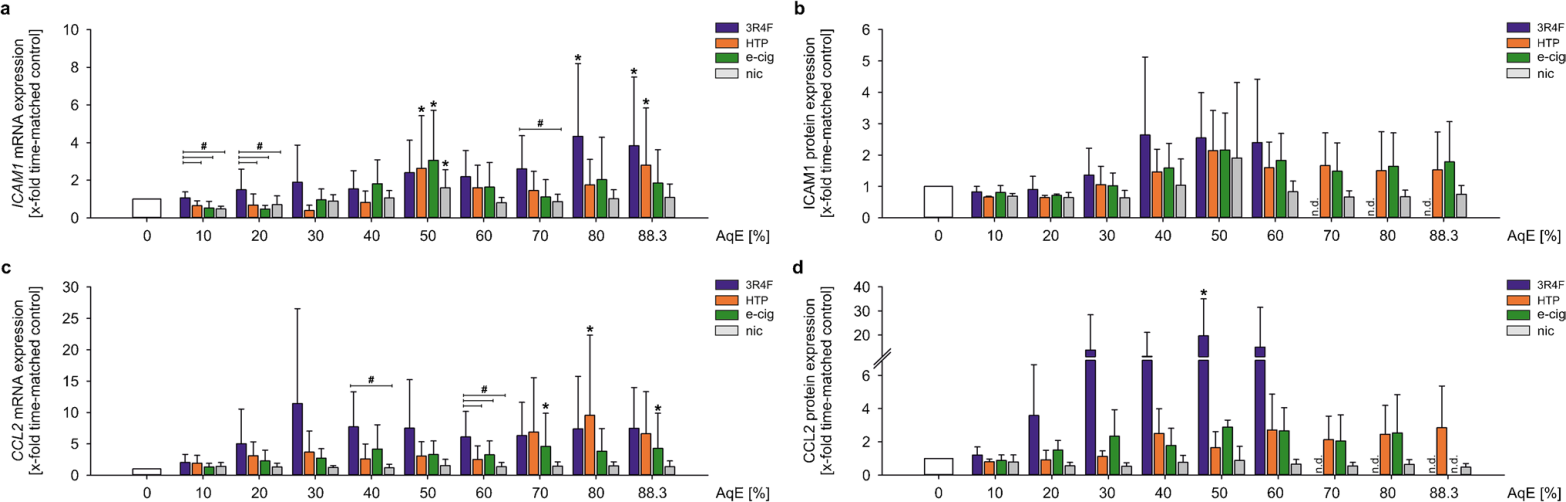
Adhesion molecule expression is induced after exposure to smoke extracts of all tested products. mRNA and protein expression of ICAM1 (**a**, **b**) and CCL2 (**c**, **d**) under static conditions were determined after dose-dependent stimulation for 24 h. Data are shown as mean (*x*-fold static time-matched controls) ± SD. Statistics: one-way ANOVA, *p < 0.05 vs. time-matched control, #*p* < 0.05, *n* ≥ 4

Attachment of monocytes to the vascular wall is a crucial step in the progression of atherosclerosis and reflects the cellular inflammatory state. This adhesion of monocytes to endothelial cells is mainly mediated by the expression of adhesion molecules on both sides, endothelial cells and monocytes. Gene expression of *ICAM1* transiently increased in response to all tested tobacco and nicotine aqueous extracts in THP-1 cells (Fig. 3a). Additional analyses of ICAM1 protein expression in whole cell lysates revealed dose-dependent increases after exposure with aqueous extracts of tobacco and nicotine products, however, due to high variability without reaching statistical significance. Due to a major reduction of cell viability under 3R4F stimulation, ICAM1 protein expression could not be determined after incubation with 70 % and higher dosages of 3R4F. In contrast, pure nicotine treatment showed no effect on ICAM1 after 24 h of stimulation (Fig. 3b).

In the progression of atherosclerosis, several pro-atherosclerotic and pro-inflammatory cytokines are responsible for the induction of local inflammation and the recruitment of immune cells. One of these markers is chemokine CCL2/MCP1. In this study, *CCL2* gene expression was upregulated after treatment with 3R4F in THP-1 monocytes after 24 h, compared to nicotine treatment. Alternative tobacco and nicotine products induced *CCL2* gene expression at higher AqE concentrations (≥ 70%). Pure nicotine did not result in a regulation of *CCL2* (Fig. 3c). On the protein level, CCL2 was also significantly upregulated after treatment with 3R4F in a dose-dependent manner. Stimulation with HTP, e-cig, and nic showed the same trend without reaching statistical significance (Fig. 3d). CCL2 protein expression could not be determined for 3R4F stimulation with 70% and higher dosages.

### Treatment with AqEs of different tobacco products regulate pro-inflammatory signalling pathways

Other important mediators are the pro-inflammatory cytokines interleukin 1 beta (IL1B), chemokine (C-X-C Motif) ligand 8 (CXCL8, also known as interleukin 8 (IL8)), and the tumour necrosis factor (TNF).

**Fig. 4:**
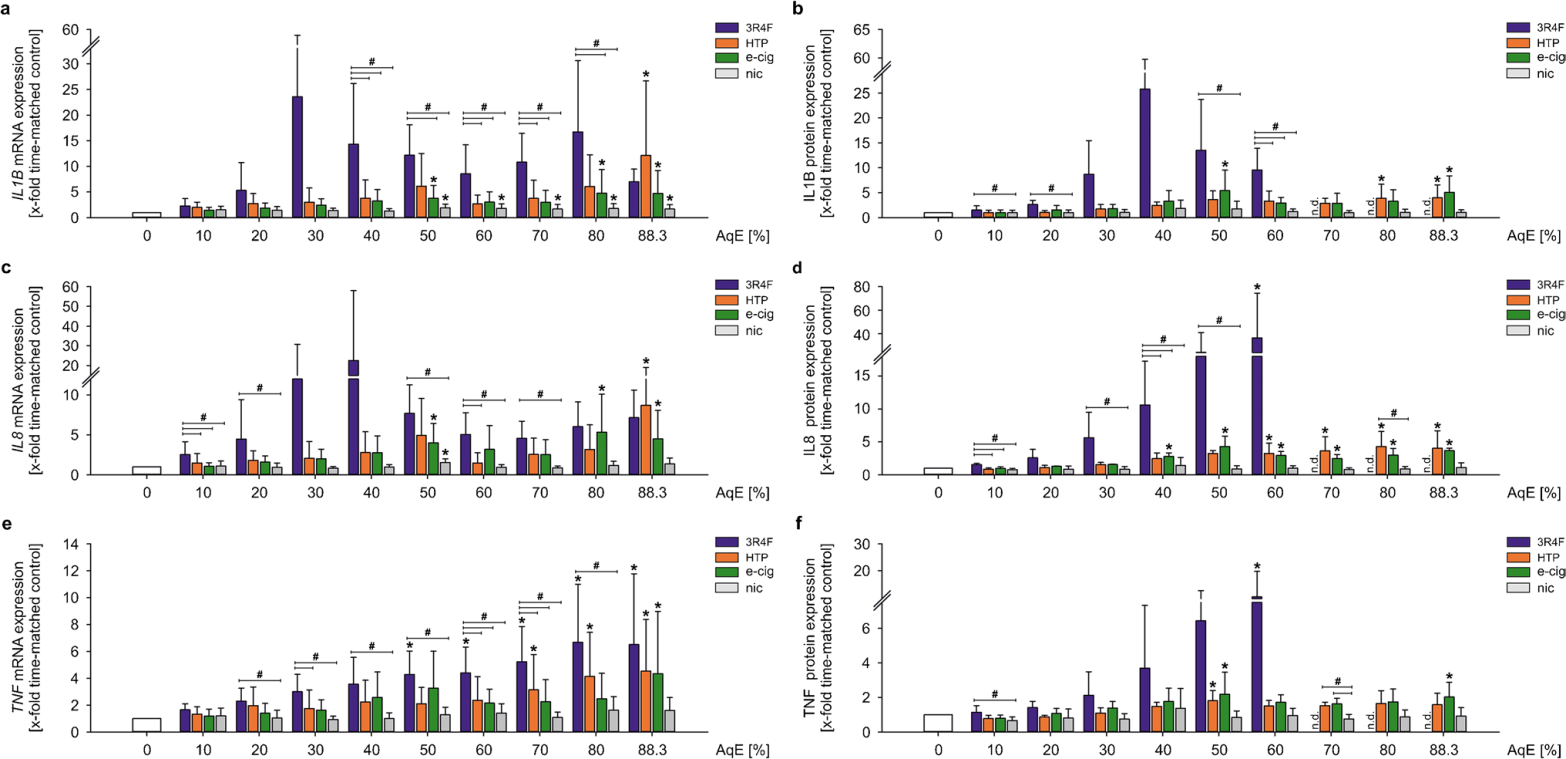
Treatment with AqEs of different tobacco products regulate pro-inflammatory signalling pathways. mRNA and protein expression of IL1B (**a**, **b**), IL8 (**c**, **d**), and TNF (**e**, **f**) under static conditions were determined after dose-dependent stimulation for 24 h. Data are shown as mean (*x*-fold static time-matched controls) ± SD. Statistics: one-way ANOVA, *p < 0.05 vs. time-matched control, #*p* < 0.05, *n* ≥ 4

Stimulation of THP-1 cells with AqEs of all tobacco/nicotine products upregulated IL1B mRNA as well as protein expression (Fig. 4a, b). Nicotine treatment also increased *IL1B* mRNA expression after a 24-h stimulation. IL8 expression showed comparable results as IL1B after stimulation with aqueous smoke extracts of all tobacco/nicotine products. 3R4F, HTP, and e-cig induced IL8 mRNA and protein expression. Nicotine treatment had only a minor effect on IL8 (Fig. 4c, d). In Fig. 4e, f, a dose-dependent induction of TNF mRNA and protein expression after stimulation with 3R4F, HTP, and e-cig is shown for 24 h. Nicotine treatment instead showed no effect on TNF expression.

Overall, the expression pattern of the investigated pro-inflammatory cytokines indicates a delayed induction under NGP treatment in comparison to 3R4F stimulation.

## Discussion

Smoking increases mortality as one of the most important, but also the single most preventable risk factor for cardiovascular diseases. The use of next-generation tobacco and nicotine products (NGP), such as the use of e-cigarettes (e-cig) and heated tobacco products (HTP), is an emerging trend in recent years. Often, considered a “safer alternative” to conventional cigarettes, little is known about their actual potentially harmful effects on the vascular endothelium. Therefore, these effects should be carefully investigated. In this study, we investigated the effect of traditional cigarettes versus next-generation tobacco and nicotine products (NGP) on human monocytes in vitro. To get detailed information about the actual deleterious impact of NGPs, this study analysed parameters of cell viability, oxidative stress, and the inflammatory state of monocytes after exposure to different smoking products.

Human THP-1 monocytes were used as a model for mimicking the function and regulation of monocytes in the vasculature. The monocyte cell line THP-1 is a well-accepted model for mimicking the function and regulation of monocytes in the vasculature [21, 58]. One of our previous studies compared the impact of cigarette smoke extract from conventional 3R4F reference cigarettes on THP-1 monocytes versus fresh isolated primary human monocytes [9]. As a result, we showed comparable results between both cell types. Based on these findings, the homogeneous genetic background, high growth rate, and unlimited availability of THP-1 cells, we recommended the use of THP-1 cells for screening assays.

Determination of cell viability is a common key parameter for product screening and gives first results about cytotoxicity of the tested compounds. Based on our own previous testing with primary endothelial cells and monocytes, different cigarette smoke extract dosages, and various time points, we decided to use ATP-based cell viability assays for investigation of the effects of next-generation tobacco and nicotine products in this study [9, 26, 27]. As a result, alternative nicotine/tobacco products seem to have minor cytotoxic effects on THP-1 monocytes compared to 3R4F AqE. For 3R4F, significant dose-dependent reduced cell viability has been shown for dosages ≥ 30%. Other studies showed similar conclusions by comparing traditional cigarettes and NGP regarding cytotoxicity [7]. Similar concentrations of AqE were applied in recent studies investigating the induction of a pro-inflammatory phenotype and cell death of endothelial cells [31, 57, 78].

Key elements in the pathogenesis of endothelial dysfunction and atherogenesis are oxidative stress and inflammatory status. Cigarette smoke itself contains different populations of free radicals causing various negative vascular effects. In addition, cigarette smoking-induced intracellular ROS production leads to oxidative stress [6, 59, 75], which furthermore activates the vascular endothelium by promoting inflammatory gene expression and consequently the production of pro-atherogenic adhesion molecules and cytokines [20, 77]. As we could show previously, cigarette smoke induces NRF2 antioxidative defence system in endothelial cells and human monocytes as well as in vivo [9, 26]. While *NRF2* expression itself remains unchanged, the expression of its target genes *HMOX1* and *NQO1* were induced in a dose-dependent manner. Under stimulation with NGP, human monocytes typically showed a lower and rightwards shifted activation of the cellular oxidative stress response. Recent studies investigated the effects of NGPs on cellular oxidative stress and the NRF2 system in various cellular systems [67, 68]. They consistently revealed a reduced and often delayed onset of this antioxidative cellular defence system comparing NGP use and traditional smoking. This is in line with our own findings in this study.

Reactive oxygen species play a crucial role in the progression of atherosclerosis. Particularly the production of superoxide by NOX2, a marker of oxidative stress in monocytes contributes to the development of endothelial dysfunction and atherosclerosis [38]. Therefore, we analysed the effects of 3R4F, e-cig, and HTP on NOX2 in human monocytes. Interestingly, stimulation with e-cig as well as with HTP induced significantly NOX2 mRNA expression at high AqE concentrations compared to 3R4F. In contrast to the other investigated markers, NOX2 is only regulated to a minor degree by 3R4F, but seems to be induced by high dosages of NGP treatment. Our study shows in this respect contrary results compared to previous literature. In these studies, it has been shown that cigarette smoke increases NOX2 expression and superoxide production by NOX2 in vitro and in vivo [36, 64]. Also, smoking e-cigarettes as well as traditional cigarettes led to a significant increase in NOX2 levels in human volunteers with a lower impact of e-cigarettes [10].

Cigarette smoking results in a chronic systemic inflammatory response [4, 52]. Several pro-inflammatory key markers are essential for the adhesion of monocytes to the endothelium, which is a crucial step in vascular inflammation and the progression of atherosclerotic lesions [15]. One of our previous in vitro studies documented an increased monocyte adhesion to endothelial cells treated with 3R4F in vitro [26]. This is consistent with other studies showing similar results after 3R4F stimulation [54, 56]. Several other in vitro and in vivo studies have previously demonstrated increased monocyte adhesion to endothelial cells in response to smoking [18, 19, 35]. These functional data could have been shown to be related to an enhanced mRNA expression of several inflammatory marker genes as adhesion molecules and cytokines. Nevertheless, there are studies showing increased expression of inflammatory markers in response to conventional cigarettes as well as to alternative tobacco products [46, 59, 75]. The pro-inflammatory phenotype of monocytes has been determined in this study by expression analysis of ICAM1, CCL2, IL1B, IL8, and TNF. The induction of ICAM1 mRNA and protein expression under stimulation with cigarette smoke is in line with own previous studies and existing literature associating cigarette smoking with increased monocyte adhesion mediated by enhanced adhesion molecule expression [1, 60]. Poussin et al. showed an elevated endothelial ICAM1 expression after treatment with supernatants from 3R4F-stimulated MM6 monocytes, indicating an inflammatory profile of human monocytes that were exposed acutely to smoking extracts of 3R4F reference cigarettes [54]. CCL2, a monocyte-specific surface marker that induces local inflammation and accelerates atherosclerosis, showed an upregulation on mRNA and protein levels for only 3R4F but not consistently for NGP treatment. Studies show increased CCL2 in human endothelial cells by extracts of smokeless tobacco [24] and that this activation of CCL2 might be involved in the CSEaq-mediated monocyte adhesion to endothelial cells [28]. Therefore, CCL2 seems to be a solid key parameter for analysing the cellular inflammatory response after treatment with various tobacco products. Our data suggest a later onset of an inflammatory phenotype under stimulation with e-cig and HTP in comparison with 3R4F in human monocytes. This higher deleterious potential of 3R4F could be confirmed by investigating the expression of IL1B, IL8, and TNF in human monocytes. A dose-dependent increase in mRNA and protein levels of these markers could be detected, whereas NGP treatment had only minor effects. This stands in line with a study from Walters et al. showing the smoke-induced release of IL1B, IL8, and TNF in human monocytes [74] and further supported by a study from Yang et al. revealing an induction of IL8 and TNF expression in MonoMac6 monocytes after CSEaq stimulation [79].

An important aspect which needs to be carefully considered while interpreting and comparing data from smoke extract treatments is the normalization of used AqE concentrations. In the corresponding literature, various normalization factors, e.g. number of puffs, percentages, or nicotine amounts are used for the application of smoke extracts in vitro and in vivo [23, 55, 66, 76]. In order to compare effects in different smoking studies, it is mandatory to state and consider the detailed product specifications. In the current study, all types of AqE were produced using the same conditions and parameters (HCI smoking regime). Percentage-matched stimulations were performed resulting in equal dilutions of the different products with known nicotine amounts and minimized variations, which can be compared which each other.

Next-generation tobacco and nicotine products (NGP) are often considered a “safer alternative” to conventional smoking which makes their actual biological impact currently of substantial interest. Various data from in vitro, animal, and human clinical studies have been published. Most of these studies show similar results about the biological impact of NGPs in relation to conventional cigarettes. This corroborates the relevance of in vitro approaches in research to characterize the molecular and functional effects of test compounds under simplified and defined in vitro conditions mimicking in vivo conditions.

## Conclusion

This study suggests that in vitro stimulation of human monocytes with conventional cigarette leads to onset of antioxidative and pro-inflammatory mechanisms at lower doses, whereas treatment with alternative smoking products leads to reduced activation of the analysed study parameters: cell viability, cellular oxidative stress response, and inflammatory state. Exposition to NGP typically shifted the response rightward to higher doses compared to 3R4F. Therefore, this study protocol can be considered a valid model system for the assessment of next-generation tobacco and nicotine products.

## Data Availability

All datasets as well as relevant information about methods and used materials are documented, saved electronically, and managed by Sindy Giebe and Coy Brunssen at Division of Vascular Endothelium and Microcirculation, Department of Medicine III, University Hospital Carl Gustav Carus Dresden, Technische Universität Dresden, Dresden, Germany. For any requests, please contact the corresponding author.
